# Differences between the normal and perceived appropriate portion sizes of discretionary foods

**DOI:** 10.1038/s41430-025-01569-2

**Published:** 2025-01-21

**Authors:** Qingzhou Liu, Margaret Allman-Farinelli, Anna Rangan

**Affiliations:** 1 School of Life and Environmental Sciences, Faculty of Science, The University of Sydney, Sydney, NSW 2006 Australia; 2 Charles Perkins Centre, The University of Sydney, Sydney, NSW 2006 Australia; 3 Discipline of Nutrition and Dietetics, Susan Wakil School of Nursing and Midwifery, Faculty of Medicine and Health, The University of Sydney, Sydney, NSW 2006 Australia

**Keywords:** Nutrition, Public health

## Abstract

**Background:**

There are limited data on the amount of discretionary foods that people normally consume and consider as appropriate at one eating occasion. This study aimed to provide an overview of the range and assess differences of the ‘normal portion size’ and ‘perceived appropriate portion size’ of energy-dense nutrient-poor discretionary foods among consumers aged 18–65 years.

**Methods:**

To measure normal and perceived appropriate portion sizes, a validated online image-series questionnaire consisting of eight successive portion size options for 15 discretionary foods was completed at two timepoints. Quantile regression models were used to estimate the ranges (lower boundary at 17^th^ percentile, upper boundary at 83^rd^ percentile) of normal and perceived appropriate portion sizes selected by two thirds of the study population. Models were adjusted for the effects of potential influencing factors including biological sex, age, usual physical activity level, cooking confidence, socio-economic status, body mass index, and baseline hunger levels.

**Results:**

A final sample of 295 participants were included in the analysis (51% females, mean age 39.5 ± 14.1 years). The normal portion sizes were significantly higher than the perceived appropriate portion sizes across all test foods, with the effects of sex, age, and BMI being significant for some foods.

**Conclusion:**

The finding suggests that consumers would normally consume a portion size that was larger than what they perceive to be appropriate for discretionary foods. The estimated lower and upper boundaries would be valuable for the development of pragmatic public health messages to empower consumers towards better portion control.

## Introduction

Food serving sizes have increased considerably over time [1], with large servings and packages commonly available and attractively presented in the current food environment [2–4]. Constant exposure to larger sizes can result in consumers considering these as the new ‘normal’, a phenomenon commonly termed ‘portion distortion’ [5, 6]. This can result in excessive consumption, additional total energy intake, and excessive weight gain long term [6–8]. Large serving sizes of energy-dense, nutrient-poor discretionary foods, defined as “foods and drinks that are high in saturated fats, added sugars, added salt and/or alcohol and should be consumed sometimes and in small amounts” [9], are especially concerning. Potential public health strategies to control portion sizes of discretionary foods have been proposed, for example, sugar taxes [10]; setting a ‘cap’ rule policy to restrict supersize options [11]; providing a range of smaller serving sizes [12, 13]; and making smaller sizes the default option [12, 13]. Laboratory studies have noted positive effects when providing single smaller serving sizes on reducing the intended or actual intake of discretionary foods. However, the benefits of more extreme serving size reductions (that is, multipacks versus a single larger pack) are still controversial [13]. It should be noted that serving sizes that are considered as being ‘too small’ potentially trigger additional intake through consuming further servings and compensatory eating at later meals [14, 15]. To avoid unintended consequences, the proposed “norm range model” suggests a range of portion sizes may be considered as normal; offering serving sizes within, but at the lower end, of this range might reduce intakes unconsciously [16, 17]. Nevertheless, there is a lack of knowledge and limited data on normal portion sizes (described as the portion size people would usually or typically consume at one sitting) versus perceived appropriate portion sizes (described as the portion size people consider to be an appropriate amount to be consumed at one sitting) for common discretionary foods [17, 18]. Such understanding is necessary to inform the development of practical public health messages and campaigns to nudge consumers towards more appropriate portion size selections [17–19].

The complexity of portion size selections has been acknowledged, and is likely to be highly variable depending on multiple external and internal factors [15, 17, 18, 20]. For example, food presentation such as package size or a food unit (that is, one burger) has also been identified as an important external moderator [21]. Estimation of a normal portion size may be smaller when exposed to a single smaller package than a single larger package prior to eating [21]. This might be due to unit bias, a tendency for consumers to consider one food unit or package as the reasonable amount to consume [6, 13, 22]. The context of eating is another influencing factor; people’s eating behaviour seems to be guided more by personal norms in home settings when eating by self, whilst social norms might have a more salient impact when dining out with a group of other people [20, 23–25]. Moreover, internal moderators such as biological sex, age, and hunger status have been shown to influence the selection of normal portion sizes [20, 26–29]. Males (versus females), younger adults (versus older), and participants who fasted overnight (versus those feeling full) were shown to choose a larger amount of food as their normal portion size. Further high-quality evidence is needed to confirm these findings across a broader range of discretionary food types, for example, mid-meal snacks versus fast food as main meals [20, 26–29].

The process of conceptualising normal and perceived appropriate portion sizes has not been well studied. Normal portion sizes may reflect consumers’ habitual intake based on their past experiences [20], whilst the perceived appropriate portion sizes seem to be more closely related to social norms in terms of ‘the expected amount to be consumed according to others’ [20, 25]. However, inconsistent definitions of these concepts have been used, and some studies regarded the ‘normal’ and ‘appropriate’ portion size as alternative terms [17, 30]. Currently, there is scant data on the extent to which the normal and perceived appropriate portion sizes overlap or differ from each other, as well as whether people “normally” consume more than the amount they consider as appropriate.

Therefore, the aims of this study were to 1) estimate the ranges and assess differences between ‘normal portion sizes’ and ‘perceived appropriate portion sizes’ for commonly consumed discretionary foods among Australian adults aged 18–65 years in home settings, by using a validated portion size questionnaire; and 2) assess the variations of normal and perceived appropriate portion sizes across age (18–30, 31–50, 51–65 years old) and biological sex subgroups, and investigate the effect of other potential influencing factors. For the purpose of this study, the normal portion sizes were defined as the portion size people would normally consume at a single eating occasion; the perceived appropriate portion sizes were defined as the portion size people consider as an appropriate amount to be consumed at a single eating occasion.

## Methodology

### Recruitment

A sample of Australian consumers aged 18–65 years was recruited through online advertisements via social media sites, and the distribution of physical flyers around the university and in local communities. An online screening questionnaire excluded participants who did not meet the following criteria: aged between 18–65 years, living in Australia, fluent in English, no current or previous diagnosis of an eating disorder. Due to the preliminary nature of this study, there was insufficient information for power analysis as the effect size and expected variability could not be estimated. There is no specific guidance on sample size estimation for portion size related outcomes in consumer studies [31], a target sample size of 300 participants from the general Australian population, with a quota of 50 for each age (18–30, 31–50, 51–65 years) and sex group was considered appropriate based on reviews of similar descriptive dietary assessment studies on portion sizes [20, 32–35]. The sampling quota was set using the build-in function in Qualtrics (Provo, UT, USA, 2022).

### Survey design

A repeated cross-sectional design was used. Participants were required to complete the online survey twice, at least one week apart (November 2022 to January 2023). The survey was developed using Qualtrics, consisting of demographic questions and an image-series questionnaire to assess the normal and perceived appropriate portion sizes. The demographic questions collected information on participants’ biological sex, age, postcode of home address, self-reported height and body weight, usual physical activity level (PAL), education level, cooking confidence, and hunger level. Details of the demographic questions are attached as supplementary material (Supplementary Appendix 1).

The image-series questionnaire used in the survey was previously validated in the population of interest and showed good agreement when compared with real foods [36]. In current study, the questionnaire consisted of two identical sections for the normal and perceived appropriate portion sizes. All settings were at home, eating alone, or with family. In accordance with the latest national nutrition survey [36, 37], 15 commonly consumed discretionary foods and drinks were selected: six sweet and savoury snacks (M&Ms, chocolate bars, chocolate blocks, sweet biscuits, savoury biscuits and potato crisps); four cakes (layered cake, caramel slices, muffins, and banana bread) as mid-meal snacks; five fast foods (pizza, hot chips, and nuggets) as main meals or side dishes, and sugar-sweetened beverages (SSB; cola, in glasses or cups, and in bottles or cans) as between-meal drinks.

**Fig. 1:**
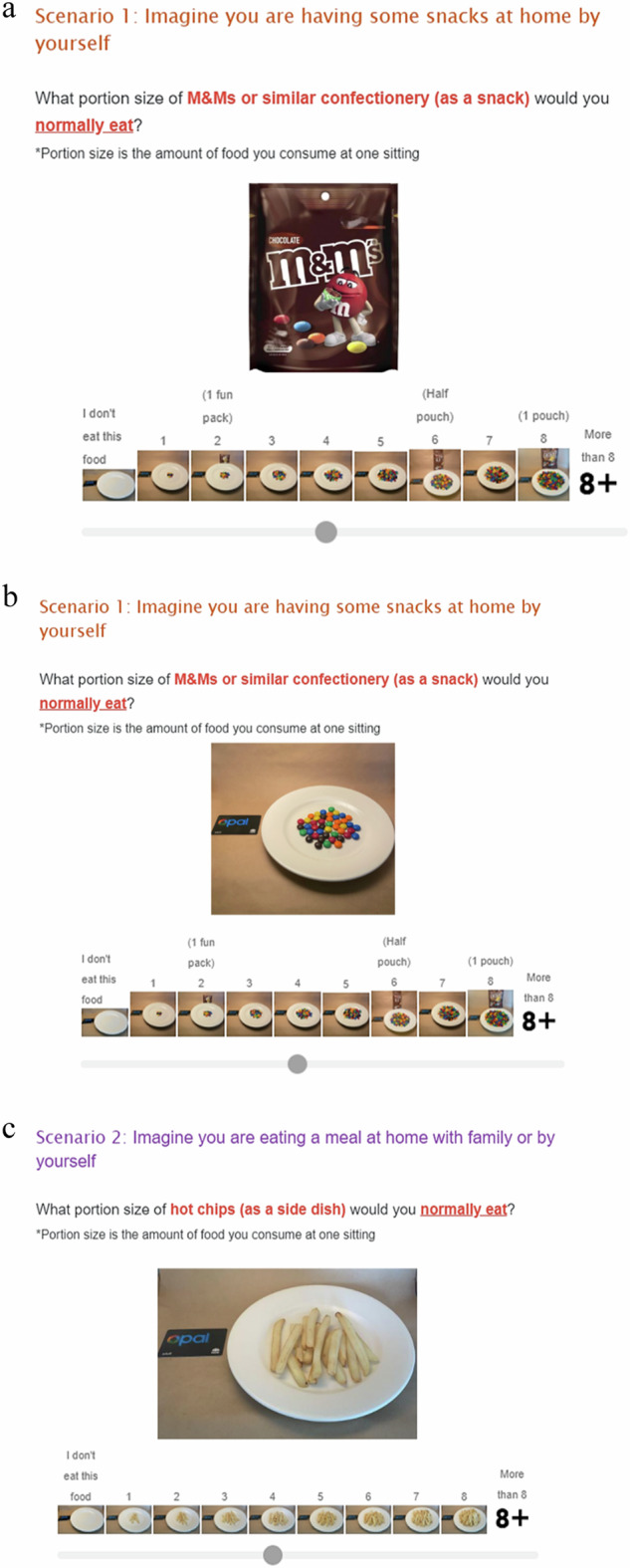
Examples of survey questions. **a** Example of the original package used as the cover photo to orient the participants to each food. **b**, **c** Examples of enlarged images for easier viewing when participants moved the cursor along the scale to their corresponding or nearest selection.

For each test food, eight successive images of portion size options were displayed corresponding with the sliding scale question, “What portion size of (the test food and context; for example, crisps as a mid-meal snack at home) would you normally eat?” or “What portion size of (the test food and context) would you consider as an appropriate amount to eat for yourself?”. The sliding scale was labelled from smallest ‘1’ to largest ‘8’ with the additional selections of ‘0 – I do not eat this food’ and ‘9 – greater than the largest option displayed’. An image of the original package was used as the cover photo to orient the participants to each food (for example, a packet of M&Ms, Fig. 1a). The selected images would become enlarged for easier viewing when participants moved the cursor along the scale to their corresponding or nearest selection (for example, snack and fast food displayed on plate, Fig. 1b, c). The definition of portion size as ‘the amount of food you consume at one sitting” was present for each question. The presentation order of the two sections (normal and perceived appropriate portion size), as well as the order of test foods displayed within each section were randomised using Qualtrics built-in randomiser. Further details of image-series tool development and validation are described in Liu et al. [36].

### Study procedure

Eligible participants who passed the screener and provided informed consent were able to access the demographic questionnaire that was built into the first survey. Basic participants characteristics information was collected, participants were required to create a username to link their answers between the two surveys in an anonymous manner. This was followed by the image-series questionnaire to assess the normal and perceived appropriate portion sizes. The invitation email with an access link to the second survey was sent one week after completion of the first survey. The second survey contained the identical image-series questionnaire but without the demographic questions; participants were required to report their username (or email address if username forgotten) and hunger level before they could proceed to the questions.

This study was approved by the Human Ethics Committee of the University of Sydney (ethics approval number 2022/147). Participants received a small compensation voucher ($10 AUD supermarket voucher) after completing both surveys. The study protocol was registered a priori on the Open Science Framework (OSF registration osf.io/xef3h).

### Data analysis

The quality of collected data was closely monitored throughout the data collection process, further details are attached as supplementary material (Supplementary Appendix 1).

Descriptive analysis was undertaken to describe participant characteristics. Due to small numbers, participants who responded as “prefer not to say” and “other” for sex (*n* = 3), were excluded from final sample for data analysis. For each food, data were excluded if participants reported not consuming a particular food or drink item in both surveys.

Quantile regression analysis was conducted to estimate the normal and perceived appropriate portion sizes at the 50^th^ quantile (median), and 17^th^ (lower boundary) and 83^rd^ (upper boundary) percentiles to establish the ranges. This range was designed to represent portion size selections of two thirds of the study population. A quantile regression model was established per test food, resulting in 15 models in total. Within the model, the data from both times of the survey (coded as “T1” and “T2”) and the measures of normal and perceived appropriate portion sizes (coded as “normal” and “appropriate”) were included. The input variables were the portion size image options selected by participants (between 0 and 9); the output variables were the predicted values after adjusting for all potential influencing factors, and the effect sizes (coefficients) of included fixed factors.

The measures of normal and perceived appropriate portion sizes were included as a fixed factor for testing the differences. The fixed effects of other potential influencing factors including sex, age (by three subgroups; 18–30, 31–50, 51–65 years), BMI (calculated from height and weight data, in kg/m^2^), cooking confidence (total scores), PAL (by four categories), baseline hunger level (visual analogue scale 0–100), were also adjusted in the model. Participant ID was used as a random effect to account for repeated measures per participant.

After running all the models, the output variables for each test food were converted into grams (or millilitres for beverages) according to the corresponding portion size weights as the last step. Descriptive analyses were performed using IBM SPSS v28 (IBM, Armonk, NY, USA, 2021), quantile regression models were performed using “lqmm” package in R statistical software version 2023.10.30 (R Core Team, Vienna, Austria, 2023) [38]. Multiple comparisons of quantile regression models were adjusted using the Holm’s sequential Bonferroni procedure as this method is more suitable to adjust for familywise error for comparisons that are not independent [39].

## Results

### Participants’ characteristics

A total of 692 subjects passed the screener questionnaire and provided consent, of these 407 subjects completed the first survey. A final sample of 295 subjects (150 females, 145 males) were included for analysis (Table 1). The mean age of participants was 39.5 years, with 42.0% of participants within the healthy weight range and 55.2% were classified as overweight or obese. The majority of participants were university educated (74.7%) and lived in a higher SES area (76.7%) based on their self-reported residential postcodes. For baseline hunger levels at two time points, most participants reported not feeling hungry, with median hunger 27 out of 100 in the first survey and 28 of 100 in the second survey.

**Table 1:** Participants’ characteristics (*n* = 295).

**Age, years, mean (SD)**	39.5 (14.1)
18–30, n (%)	98 (33.2)
31–50, n (%)	99 (33.6)
51–65, n (%)	98 (33.2)
**Biological sex, females,** ***n*** **(%)**	150 (50.9)
**BMI, kg/m**^**2**^**, mean (SD)**	27.2 (7.0)
Underweight, *n* (%)	8 (2.7)
Within healthy weight range, *n* (%)	124 (42.0)
Overweight, *n* (%)	91 (30.8)
Obese, *n* (%)	72 (24.4)
**Education level,** ***n*** **(%)**	
High school or below	55 (18.7)
Diploma equivalent	49 (16.6)
University	191 (74.7)
**Socio-economic status (SES),** ***n*** **(%)**	
Lower	67 (23.3)
Higher	221 (76.7)
**Physical activity level (PAL),** ***n*** **(%)**	
Sedentary	55 (18.6)
Lightly active	137 (46.4)
Moderately active	82 (27.8)
Very to extremely active	21 (7.1)
**Cooking confidence,** ***n*** **(%)**	
Low	112 (38.0)
High	183 (62.0)

### Ranges of normal and perceived appropriate portion sizes

The median percentile and ranges (17^th^–83^rd^ percentiles) of normal and perceived appropriate portion sizes were grouped into three discretionary food categories, and presented by sex and age subgroups in Table 2.

**Table 2:** Quantile regression analysis (17^th^, 50^th^, 83^rd^ percentiles) for normal and perceived appropriate portion sizes^a^ of discretionary foods, in gram and millilitres (beverages), by biological sex and age groups.

	*Females*						*Males*					
	*Range of normal portion sizes* Median (17^th^–83^rd^ percentile)			*Range of perceived appropriate portion sizes* Median (17^th^–83^rd^ percentile)			*Range of normal portion sizes* Median (17^th^–83^rd^ percentile)			*Range of perceived appropriate portion sizes* Median (17^th^–83^rd^ percentile)		
	18–30	31–50	51–65	18–30	31–50	51–65	18–30	31–50	51–65	18–30	31–50	51–65
Sweet and savoury snacks												
Chocolate bar	45 (21–52)	44 (21–51)	39 (20–46)	41 (20–48)	39 (20–46)	35 (18–42)	52 (23–59)	51 (23–58)	47 (22–53)	48 (22–55)	46 (22–53)	42 (20–49)
Chocolate blocks	34 (24–45)	37 (28–51)	32 (22–43)	31 (22–42)	35 (25–46)	29 (19–40)	39 (30–54)	43 (33–60)	37 (27–49)	36 (27–49)	40 (30–55)	34 (24–45)
M&Ms	29 (13–46)	34 (16–53)	19 (11–40)	19 (10–39)	28 (13–45)	16 (8–34)	32 (14–50)	37 (17–57)	21 (12–43)	20 (11–42)	31 (14–49)	18 (9–37)
Sweet biscuits	32 (25–49)	31 (24–49)	28 (22–46)	28 (21–45)	27 (20–45)	24 (17–42)	36 (29–54)	35 (29–53)	33 (26–50)	32 (25–50)	31 (25–49)	29 (22–46)
Savoury biscuits	43 (23–59)	46 (24–61)	30 (16–50)	34 (19–53)	38 (20–55)	23 (12–42)	48 (25–62)	51 (29–64)	35 (19–44)	40 (21–57)	43 (23–59)	27 (15–47)
Crisps	35 (21–44)	37 (23–46)	31 (17–40)	29 (16–39)	32 (18–41)	26 (12–35)	39 (26–49)	42 (28–61)	36 (22–45)	34 (21–44)	36 (23–46)	31 (17–40)
Cakes												
Banana bread	154 (93–172)	139 (84–164)	125 (76–157)	144 (87–166)	129 (78–159)	115 (69–148)	167 (112–185)	160 (99–177)	149 (90–170)	162 (102–179)	153 (92–171)	139 (84–164)
Caramel slices	54 (42–87)	43 (34–68)	41 (32–64)	49 (39–80)	40 (32–63)	38 (29–59)	61 (47–98)	49 (39–79)	45 (36–73)	56 (44–91)	45 (36–72)	42 (34–66)
Layered cake	92 (62–123)	84 (56–118)	68 (42–105)	81 (53–116)	74 (47–110)	59 (32–97)	102 (73–135)	95 (65–126)	79 (51–114)	92 (63–124)	85 (56–118)	69 (42–106)
Muffin	103 (45–135)	91 (38–124)	79 (31–113)	96 (41–128)	83 (33–117)	71 (26–106)	120 (59–152)	109 (49–141)	97 (42–130)	113 (42–145)	102 (44–134)	90 (37–123)
Fast foods and sugar-sweetened beverages (SSB)												
Pizza	260 (194–309)	215 (151–263)	182 (118–229)	236 (172–286)	193 (129–241)	161 (99–207)	324 (256–365)	279 (212–327)	243 (179–293)	302 (233–346)	256 (190–306)	221 (157–270)
Hot chips	105 (74–133)	103 (72–130)	83 (56–110)	95 (65–121)	93 (63–119)	76 (46–98)	130 (95–166)	128 (93–164)	108 (77–137)	119 (86–151)	117 (84–149)	97 (67–122)
Nugget	139 (95–214)	124 (74–186)	101 (52–156)	129 (81–195)	112 (59–166)	87 (43–146)	153 (116–241)	138 (95–214)	121 (69–179)	143 (102–223)	129 (80–194)	108 (56–160)
SSB cup/glass (mL)	229 (124–297)	263 (158–331)	237 (132–305)	215 (109–282)	249 (144–316)	223 (117–290)	287 (181–355)	321 (216–389)	295 (189–362)	273 (167–340)	307 (201–374)	281 (175–347)
SSB bottle/can (mL)	238 (154–276)	243 (159–281)	220 (135–262)	219 (135–262)	225 (141–266)	201 (109–246)	283 (208–329)	287 (213–336)	268 (189–307)	268 (189–307)	273 (195–313)	253 (171–290)

^a^Quantile regression models at 17^th^ (lower boundary), 50^th^ (median), and 83^rd^ (upper boundary) percentiles were established per test food to estimate the ranges of normal portion sizes (described as the amount people would usually or typically consume at one sitting) and perceived appropriate portion sizes (described as the amount people consider as appropriate to consume at one sitting).

### Comparison of normal and perceived appropriate portion sizes

Differences between the normal and perceived appropriate portion sizes were investigated (Table 3). After adjusting for all potential influencing factors, the normal portion sizes were found to be significantly higher than the perceived appropriate portion sizes for all 15 test foods (*p* < 0.01). The effect sizes varied from 0.15 to 0.52 across foods, indicating that participants selected a fifth to half an option smaller (along the scale) as their perceived appropriate portion sizes compared with their normal portion sizes.

**Table 3:** The effect sizes from quantile regression models indicating differences between normal and perceived appropriate portion sizes, by food type (*n* = 15).

Test food	Effect sizes^a^		*P*-values^b^
	*17*^*th*^	*83*^*rd*^	
Chocolate bar	0.19	0.19	<0.001*
Chocolate blocks	0.24	0.24	0.004 *
M&Ms	0.43	0.43	<0.001*
Sweet biscuits	0.29	0.30	<0.001*
Savoury biscuits	0.31	0.32	<0.001*
Crisps	0.52	0.50	<0.001*
Banana bread	0.19	0.19	0.01 *
Caramel slices	0.23	0.23	<0.001*
Layered cake	0.29	0.29	<0.001*
Muffin	0.15	0.15	0.007 *
Pizza	0.33	0.33	<0.001*
Hot chips	0.38	0.37	<0.001*
Nugget	0.24	0.24	<0.001*
SSB^c^ cup/glass	0.20	0.20	0.01 *
SSB bottle/can	0.30	0.30	<0.001*

*P-values were statistically significant after the adjustment.

^a^The effect sizes are the coefficients from quantile regression models at percentiles tau = 0.17 and 0.83; differences of normal and perceived appropriate portion sizes were tested in the models as fixed factors.

^b^*P*-values adjusted for multiple comparisons using Holm–Bonferroni method [39].

^c^SSB: sugar-sweetened beverages.

### Potential influencing factors in quantile regression models

Sex, age and BMI were found to influence normal and perceived appropriate portion sizes in selected test foods. By sex, significant differences were found for pizza, hot chips, and SSBs both in glass/cup and in bottle/can. At all three percentiles, males selected half to one portion size option larger than females (effect sizes 0.77–0.95). Across age groups, those aged 51–65 years had significantly lower normal and perceived appropriate portion sizes for layered cake and nuggets (effect sizes −0.67 to −0.78), whilst those aged 18–30 years showed significantly larger normal and perceived appropriate portion sizes than the older age groups for pizza (effect sizes −0.67 to −1.18); these differences were significant at all three percentiles. The effects of BMI on normal and perceived appropriate portion sizes were significant at the 83^rd^ percentile in 12 of 15 foods, but no effect was found at the two lower quantile levels.

Hunger, cooking confidence and SES had minimal effects on normal or perceived appropriate portion sizes. More detailed findings of the effects of potential influencing factors adjusted in quantile regression models are presented in supplementary materials (Supplementary Appendix 2).

## Discussion

The ranges of normal and perceived appropriate portion sizes of discretionary foods were estimated using a validated image-series tool. Overall, the findings suggested significant differences between normal and perceived appropriate portion sizes, with normal portion sizes being significantly higher than the perceived appropriate portion sizes across all 15 discretionary foods. We found individual demographic factors including sex, age and BMI had impacts on normal and perceived appropriate portion sizes for selected foods. Males (versus females), younger adults aged 18–30 years (versus those aged 31–65 years), and those with higher BMI reported larger normal and perceived appropriate portion sizes.

This finding is consistent with a proposed theory that the conceptualisation process of the normal and perceived appropriate portion sizes might be different [17]. Personal norms and the perception of own usual intake appear to play a major role in the formation of normal portion sizes, whereas social factors and norms such as peer influence, and cultural customs and conventions, may be more closely connected with perceived appropriate portion sizes [40, 41]. Overconsumption of palatable discretionary foods is commonly regarded as ‘inappropriate’ and lacking self-control [34], the significantly lower perceived appropriate portion sizes than normal portion sizes is likely to be associated with the tendency to behave in a socially approved manner [25, 40]. However, the overlap between the normal and perceived appropriate portion sizes observed in this study suggests they are closely linked to one another.

Regarding potential influencing factors, we observed significant positive effects of BMI at the upper boundary of normal and perceived appropriate portion sizes in most test foods, and the influence of sex and age was significant in some foods. Two previous studies noted a similar positive relationship between BMI and self-selected normal portion sizes [20, 28]. This is likely a result of larger habitual intake of discretionary food among those in the overweight and obese categories than individuals within the healthy weight range [8, 42]. One study suggested people in the obese group were aware that their personal eating norm (how much they would normally consume) was higher than their social eating norm (how much most other people would normally consume) [20]. However, the relationship between perceptions of appropriate portion size and weight status has not been well documented. It remains to be tested that whether BMI associate with both normal and perceived appropriate portion sizes, or one but not another. Previous literature has identified sex and age differences in portion size selections, with males and those in the younger age group selecting larger portion sizes than females and those in the older age group [29, 43]. In present study, the effect of sex and age was significant in a few test foods. This may be attributed to differences in test food selection and characteristics, considering one study that included various food and meal types only found significant sex differences on selected usual portion sizes for main meals and side dishes, but not snack foods [43]. Importantly, hunger level did not appear to influence normal and perceived appropriate portion sizes in this study. It could be due to the way questions were formulated; participants were asked to report what they would do at one sitting in the given situation, rather than their usual intake or preferred portion sizes based on current state of hunger. This finding suggests consumers are likely have a clear sense of own normal and perceived appropriate portion sizes, that is, the “portion size norms” [17, 18, 25]. Such norms have been developed and learned over a person’s life course, influenced by various factors such as individual characteristics, family conventions, prior personal experiences of habitual eating, as well as the broader external socio-cultural environment [25, 44].

The median and upper boundaries of ranges of the normal portion sizes reported in this study are consistent with some existing public health recommendations [45, 46]. A recent Australian government report to guide food industry to voluntarily reduce portion sizes of discretionary foods has proposed upper limits on several discretionary foods such as sweet biscuits, confectionery, layered cakes, muffin, pizza, crumbed proteins, and chilled beverages [45]. For example, for the retail setting, the recommended serving sizes were less than 30 g for sweet biscuits, less than 50 g for a single chocolate bar, less than 90 g for muffins and layered cakes [45]. These recommended serving sizes closely align with the median percentile of the normal portion sizes in most age and sex subgroups, but upper boundaries (83^rd^ percentiles) of the ranges are considerably higher [45]. This indicates approximately half of participants exceeded these targets. Thus, changes to the current food environment would be essential to reduce the normal portion sizes to align with the recommendations and better understanding of consumers’ acceptance of serving size reductions and portion control campaigns is required [19, 41, 47]. We acknowledge that portion sizes reported in this study were specific to a single sitting whilst Australian adults were found to consume discretionary foods at more than six eating occasions on a daily basis [48]. As all questions were related to eating alone or with family at the home setting, external influences were mostly absent and social judgement is likely a lesser issue [25]. It is worth for future studies to test the potential impacts of frequency of consumption and other contextual factors (for example, eating by self, with peers and at home or social gatherings) on portion size decisions.

## Strengths and limitations

Several strengths of this study should be noted. A large sample of Australian consumers from various demographic areas were recruited, and the age and sex groups of the study population were evenly distributed. Two thirds of the recruited sample were classified in the overweight and obese category, which is representative of the population [49]. The online image-based questionnaire was easily accessible using any smart electronic device and showed good prior validity in the target population [36]. The portion size image-series was carefully designed based on available serving sizes in real-life retail environments to ensure useability. Other strengths included the clear description of normal and perceived appropriate portion sizes, with multiple potential influencing factors adjusted within the quantile regression models.

However, we acknowledge limitations exist. To minimise participation bias, the potential impacts of internal factors such as individual differences in familiarity with each food, frequency of consumption, health consciousness were not measured in the study. The chance of estimation bias exists as it is difficult to make precise judgements of portion sizes [35]. This may be more challenging for online studies as a previous study noted portion sizes reported using virtual reality were higher than the portion sizes consumed [50]. It should be recognised that most of recruited participants were from high SES regions and may be more health conscious than the general population. Other differences such as participants’ nutrition knowledge and skills, living conditions (for example, family size), and eating habits (for example, snacking frequency and missing meals) could all potentially contribute to variations in portion size estimations [19]. Future studies conducted in naturalistic environments with study samples from various population subgroups and cultural backgrounds, as well as a more complete set of influencing factors would be beneficial to extend our understanding of the role of normal and perceived appropriate portion sizes on actual portion size selection, consumption and total energy intake.

## Conclusion and implication

This study established an overview of the ranges of normal and perceived appropriate portion sizes for various discretionary foods using an at-home setting. Significantly lower perceived appropriate portion sizes than normal portion sizes were observed across all test foods, emphasising that many Australian consumers would normally consume an amount that is higher than what they regard as appropriate. The established upper boundaries of the ranges should be further tested to explore the feasibility of setting a size limit on discretionary food to reduce the availability of supersized options. The lower boundaries might be valuable for the development of a broader range of smaller serving and package size options (for example, multipacks and fun packs of snacks, individual-size fast foods), which could help nudge consumers towards lower discretionary food intake without triggering compensatory eating behaviours [15]. We acknowledge that downsizing the serving or package is only one of the many public health strategies to reduce discretionary foods portion sizes and prevent excessive intake. Other strategies including proportional pricing, food labelling, and packaging cues [19, 41], together with the active engagement and partnerships between stakeholders (for example, industry bodies, government organisation, retailers) are essential for creating a healthier food environment that encourage more appropriate portion size selections.

## Supplementary information

Supplementary material

## Data Availability

The data sets generated and analysed during the current study are not publicly available due to the data confidentiality requirements of the ethics committee but are available from the corresponding author on reasonable request and approval from the ethics committee.
