# Supplementary material for: Differences between the normal and perceived appropriate portion sizes of discretionary foods
Source: Eur J Clin Nutr. 2025 Jan 21;79(5):419–26. doi: 10.1038/s41430-025-01569-2 (PMC12069090; doi:10.1038/s41430-025-01569-2)
Supplement: Supplementary file 1 — Supplementary material [file 41430_2025_1569_MOESM1_ESM.docx]

**Supplementary material**

**Appendix 1. Details of survey design**

**Demographic questions**

The demographic questions collected information on participants’ biological sex, age, postcode of home address, self-reported height and body weight, usual physical activity level (PAL), education level, cooking confidence, and hunger level.

The socio-economic indexes for areas (SEIFA) were used to assign participants into deciles (1-10) of economic disadvantage based on self-reported residential postcodes [1], with deciles 1-5 grouped as lower socioeconomic status (SES) and deciles 6-10 as higher SES. Baseline hunger level was assessed using a validated visual analogue scale ranging from 0 to100, with 0 indicating “not hungry at all” and 100 as “extremely hungry” [2, 3]. PAL was estimated based on the frequency and intensity of usual exercise, and classified as sedentary, lightly active, moderately active, and very to extremely active [4, 5]. Cooking confidence was assessed as a marker of food literacy level using a 5-point validated Likert scale (can cook a nutritious meal; can cook a meal in a short amount of time; can cook without spending a lot of money; can follow a recipe) [6, 7]; the total score of cooking confidence equal or higher than 16 out of 20 was classified as high, otherwise as low [6, 7].

**Data quality check**

The quality of collected data was closely monitored throughout the data collection process and checked using multiple methods. When completing the questionnaire, a ‘forced response’ was required for each question, thus participants were required to click on the cursor and make their selection before proceeding forwards. Incomplete surveys, bots and repeated responses were detected using the Qualtrics embedded function for response quality [8]; these responses were excluded from the study (n=10). In addition, the responses at both time points were manually checked by the research team to exclude participants who selected the same portion size options throughout the survey (for example, if a participant selected the middle option for all questions); no responses were excluded at this stage.

**Appendix 2. Quantile regression models supplementary information**

Table S1. The effects of potential influencing factors ^a^ on normal and perceived appropriate portion sizes, by food types (n=15).

| Test foods, n ^b^ | Quantile levels | Biological sex  (female as reference) | | Age groups  (18-30 years as reference) | | | | PAL  (slightly active as reference) | | | | | | BMI | | Hunger | | Cooking confidence | |
| --- | --- | --- | --- | --- | --- | --- | --- | --- | --- | --- | --- | --- | --- | --- | --- | --- | --- | --- | --- |
|  |  |  |  | 31-50 years | | 51-65 years | | Sedentary | | Moderate | | Very to extreme | |  |  |  |  |  |  |
|  |  | *Effect sizes* | *P-values ^c^* | *Effect sizes* | *P-values* | *Effect sizes* | *P-values* | *Effect sizes* | *P-values* | *Effect sizes* | *P-values* | *Effect sizes* | *P-values* | *Effect sizes* | *P-values* | *Effect sizes* | *P-values* | *Effect sizes* | *P-values* |
| Sweet biscuits, 284 | 0.17 | 0.32 | 0.05 | -0.06 | 0.73 | -0.25 | 0.14 | -0.01 | 0.94 | 0.24 | 0.14 | 0.77 | 0.02 | <-0.01 | 0.91 | <0.01 | 0.06 | -0.07 | 0.02 |
|  | 0.83 | 0.32 | 0.05 | -0.06 | 0.73 | -0.25 | 0.14 | -0.02 | 0.94 | 0.25 | 0.14 | 0.77 | 0.01 | 0.04 | **0.005*** | <0.01 | 0.01 | -0.03 | 0.28 |
| Savoury biscuits, 273 | 0.17 | 0.21 | 0.21 | 0.12 | 0.56 | -0.50 | 0.04 | <-0.01 | 0.97 | 0.16 | 0.33 | -0.03 | 0.95 | 0.02 | 0.36 | <0.01 | 0.08 | -0.05 | 0.05 |
|  | 0.83 | 0.21 | 0.21 | 0.12 | 0.56 | -0.50 | 0.04 | <-0.01 | 0.97 | 0.16 | 0.33 | -0.03 | 0.95 | 0.05 | **0.005*** | <0.01 | 0.02 | -0.02 | 0.41 |
| Crisps, 279 | 0.17 | 0.48 | 0.02 | 0.22 | 0.42 | -0.36 | 0.14 | 0.22 | 0.56 | 0.41 | 0.11 | 0.11 | 0.76 | <0.01 | 0.92 | <0.01 | 0.67 | -0.06 | 0.04 |
|  | 0.83 | 0.50 | 0.01 | 0.22 | 0.43 | -0.36 | 0.14 | 0.22 | 0.56 | 0.41 | 0.11 | 0.12 | 0.75 | 0.07 | **0.002*** | <0.01 | 0.04 | -0.06 | 0.08 |
| M&Ms, 261 | 0.17 | 0.20 | 0.23 | 0.37 | 0.18 | -0.34 | 0.13 | 0.02 | 0.92 | 0.60 | 0.03 | 0.54 | 0.21 | <0.01 | 0.73 | <0.01 | 0.28 | -0.10 | **0.003*** |
|  | 0.83 | 0.21 | 0.22 | 0.37 | 0.18 | -0.34 | 0.12 | 0.02 | 0.92 | 0.61 | 0.03 | 0.54 | 0.21 | 0.06 | **0.003*** | <0.01 | 0.10 | -0.06 | 0.05 |
| Chocolate blocks, 283 | 0.17 | 0.43 | 0.02 | 0.28 | 0.24 | -0.21 | 0.36 | -0.19 | 0.45 | 0.11 | 0.59 | 0.75 | 0.07 | <0.01 | 0.55 | <0.01 | 0.17 | -0.04 | 0.26 |
|  | 0.83 | 0.44 | 0.02 | 0.28 | 0.24 | -0.21 | 0.37 | -0.19 | 0.45 | 0.11 | 0.60 | 0.75 | 0.07 | 0.05 | **0.009*** | <-0.01 | 0.96 | 0.01 | 0.71 |
| Chocolate bar, 266 | 0.17 | 0.31 | 0.05 | -0.06 | 0.77 | -0.25 | 0.19 | -0.23 | 0.31 | 0.05 | 0.77 | 0.38 | 0.27 | <0.01 | 0.97 | <-0.01 | 0.79 | 0.03 | 0.28 |
|  | 0.83 | 0.31 | 0.05 | -0.06 | 0.77 | -0.25 | 0.19 | -0.22 | 0.32 | 0.05 | 0.76 | 0.38 | 0.26 | <0.01 | 0.08 | <0.01 | 0.50 | <0.01 | 0.75 |
| Layered cake, 277 | 0.17 | 0.31 | 0.05 | -0.21 | 0.37 | -0.67 | **<0.001*** | -0.17 | 0.41 | 0.04 | 0.84 | 0.44 | 0.29 | <-0.01 | 0.82 | <0.01 | 0.01 | -0.05 | 0.11 |
|  | 0.83 | 0.31 | 0.05 | -0.21 | 0.38 | -0.67 | **<0.001*** | -0.17 | 0.42 | 0.05 | 0.83 | 0.44 | 0.29 | 0.03 | 0.04 | 0.01 | **<0.001*** | -0.02 | 0.57 |
| Muffin, 275 | 0.17 | 0.36 | 0.02 | -0.25 | 0.18 | -0.47 | 0.01 | -0.02 | 0.91 | 0.11 | 0.55 | 0.86 | 0.03 | 0.02 | 0.16 | <0.01 | 0.40 | -0.02 | 0.51 |
|  | 0.83 | 0.36 | 0.02 | -0.25 | 0.18 | -0.47 | 0.01 | -0.02 | 0.91 | 0.11 | 0.54 | 0.86 | 0.03 | 0.06 | **<0.001*** | <0.01 | 0.02 | <0.01 | 0.83 |
| Banana bread, 273 | 0.17 | 0.44 | 0.03 | -0.26 | 0.25 | -0.52 | 0.03 | 0.27 | 0.28 | -0.12 | 0.52 | 0.70 | 0.13 | 0.03 | 0.04 | <0.01 | 0.13 | 0.02 | 0.54 |
|  | 0.83 | 0.44 | 0.03 | -0.26 | 0.25 | -0.52 | 0.03 | 0.27 | 0.28 | -0.12 | 0.52 | 0.70 | 0.13 | 0.08 | **<0.001*** | <0.01 | **0.002*** | 0.04 | 0.30 |
| Caramel slices, 261 | 0.17 | 0.33 | 0.009 | -0.15 | 0.36 | -0.37 | 0.02 | -0.13 | 0.46 | 0.34 | 0.03 | 0.40 | 0.26 | <-0.01 | 0.85 | <0.01 | 0.40 | -0.04 | 0.02 |
|  | 0.83 | 0.33 | 0.008 | -0.15 | 0.36 | -0.36 | 0.02 | -0.13 | 0.46 | 0.34 | 0.03 | 0.40 | 0.26 | 0.04 | **<0.001*** | <0.01 | 0.02 | <0.01 | 0.77 |
| Pizza, 291 | 0.17 | 0.95 | **<0.001*** | -0.67 | **<0.001*** | -1.18 | **<0.001*** | -0.63 | **0.001*** | <0.01 | 0.96 | 0.15 | 0.77 | 0.02 | 0.12 | <0.01 | 0.56 | -0.03 | 0.31 |
|  | 0.83 | 0.95 | **<0.001*** | -0.67 | **<0.001*** | -1.18 | **<0.001*** | -0.63 | **0.001*** | 0.01 | 0.95 | 0.15 | 0.77 | 0.06 | **<0.001*** | <0.01 | **0.002*** | <-0.01 | 0.77 |
| Hot chips, 286 | 0.17 | 0.84 | **<0.001*** | -0.07 | 0.76 | -0.74 | 0.005 | 0.46 | 0.14 | <0.01 | 0.98 | 0.06 | 0.86 | -0.02 | 0.24 | <0.01 | 0.65 | -0.04 | 0.13 |
|  | 0.83 | 0.85 | **<0.001*** | -0.07 | 0.76 | -0.74 | 0.005 | 0.46 | 0.14 | <0.01 | 0.97 | 0.06 | 0.86 | 0.03 | 0.05 | <0.01 | 0.39 | <0.01 | 0.75 |
| Nugget, 247 | 0.17 | 0.35 | 0.04 | -0.36 | 0.10 | -0.78 | **0.001** | -0.08 | 0.70 | 0.22 | 0.22 | 0.46 | 0.20 | <-0.01 | 0.90 | <-0.01 | 0.35 | 0.02 | 0.33 |
|  | 0.83 | 0.35 | 0.03 | -0.36 | 0.11 | -0.78 | **0.001** | -0.08 | 0.70 | 0.22 | 0.22 | 0.46 | 0.20 | 0.05 | **<0.001*** | <0.01 | 0.62 | <0.01 | 0.87 |
| SSB bottle/can, 190 | 0.17 | 0.86 | **<0.001*** | 0.09 | 0.72 | -0.29 | 0.28 | 0.26 | 0.37 | 0.02 | 0.92 | 0.05 | 0.90 | <0.01 | 0.78 | <0.01 | 0.12 | -0.01 | 0.72 |
|  | 0.83 | 0.86 | **<0.001*** | 0.09 | 0.72 | -0.29 | 0.28 | 0.27 | 0.36 | 0.02 | 0.92 | 0.05 | 0.91 | 0.07 | **<0.001*** | <0.01 | 0.19 | <0.01 | 0.96 |
| SSB glass/cup, 197 | 0.17 | 0.77 | **<0.001*** | 0.46 | 0.06 | 0.11 | 0.67 | -0.05 | 0.91 | 0.03 | 0.90 | 0.23 | 0.55 | <0.01 | 0.66 | <0.01 | 0.04 | -0.05 | 0.09 |
|  | 0.83 | 0.77 | **<0.001*** | 0.46 | 0.06 | 0.11 | 0.67 | -0.05 | 0.91 | 0.03 | 0.90 | 0.23 | 0.55 | 0.07 | **0.004*** | 0.01 | 0.006 | -0.02 | 0.53 |

^a^ Biological sex, age and PAL as categorical data; BMI, hunger and cooking confidence as continuous data in quantile regression models.

^b^ For each food, data were excluded if participants reported not consuming a particular food or drink item in both surveys.

^c^ Multiple comparisons adjusted using Holm’s sequential procedure [9], *p-values significant**.**

References

1. Australian Bureau of Statistics. (2016) Census of population and housing: Socio-economic indexes for areas (SEIFA).

2. Flint A, Raben A, Blundell JE, Astrup A (2000) Reproducibility, power and validity of visual analogue scales in assessment of appetite sensations in single test meal studies. Int J Obes Relat Metab Disord 24(1):38-48.

3. Forbe C. (2017) Measuring satiation and satiety. Methods in consumer research. 2: Woodhead Publishing. p. 478.

4. Food and Agricultural Organization of the United Nations, editor Human energy requirements : Report of a joint fao/who/unu expert consultation : Rome, 17-24 october 2001. FAO food and nutrition technical report series, 1; 2004; Rome: Food and Agricultural Organization of the United Nations.

5. Yang YJ (2019) An overview of current physical activity recommendations in primary care. Korean J Fam Med 40(3):135-142.

6. Gaines A, Robb CA, Knol LL, Sickler SM (2014) Examining the role of financial factors, resources and skills in predicting food security status among college students. International Journal of Consumer Studies 38:374-384.

7. Utter J, Larson N, Laska MN, Winkler M, Neumark-Sztainer D (2018) Self-perceived cooking skills in emerging adulthood predict better dietary behaviors and intake 10 years later: A longitudinal study. J Nutr Educ Behav 50(5):494-500.

8. Qualtrics. (2023) Response quality Provo, UT, USA, Qualtrics [URL|.

9. Eichstaedt KE, Kovatch K, Maroof DA (2013) A less conservative method to adjust for familywise error rate in neuropsychological research: The holm's sequential bonferroni procedure. NeuroRehabilitation 32(3):693-696.
